# Prevalence and Correlates of Psychiatric Symptoms in Minimally Verbal Children and Adolescents With ASD

**DOI:** 10.3389/fpsyt.2019.00043

**Published:** 2019-02-18

**Authors:** Daniela Plesa Skwerer, Robert M. Joseph, Brady Eggleston, Steven R. Meyer, Helen Tager-Flusberg

**Affiliations:** ^1^Department of Psychological and Brain Sciences, Boston University, Boston, MA, United States; ^2^Department of Anatomy and Neurobiology, Boston University School of Medicine, Boston, MA, United States

**Keywords:** psychopathology, minimally verbal autism spectrum disorder, maladaptive behavior, children, adolescents

## Abstract

Despite many studies documenting the prevalence of various co-occurring psychiatric symptoms in children and adults with ASD, less is known about how these symptoms relate to subtypes defined by particular phenotypic features within the ASD population. We examined the severity and prevalence of comorbid symptoms of psychopathology, emotion dysregulation, and maladaptive behaviors, as well as adaptive functioning, in a group of 65 minimally verbal children (*n* = 33) and adolescents (*n* = 32) with ASD. On the *Child and Adolescent Symptom Inventory* (CASI-5), for all the symptom classifications except oppositional defiant disorder and conduct disorder, more participants in our sample showed elevated or clinically concerning severity scores relative to the general population. On the *Emotion Dysregulation Inventory (EDI)*, the mean scores for Reactivity and Dysphoria factors in our sample were lower than in the autism calibration sample, which included a large number of inpatient youth with ASD. Overall, few differences were found between the children and adolescents within this severely impaired group of ASD individuals based on clinical cutoff scores on the CASI-5 and EDI factor scores. Psychiatric comorbidities and emotion dysregulation measures were not correlated with autism symptom severity or with measures of adaptive functioning, and were largely unrelated to IQ in our sample. The number of clinically significant psychiatric symptoms on the CASI-5 emerged as the main predictor of maladaptive behaviors. Findings suggest a wide range of co-occurring psychopathology and high degree of maladaptive behavior among minimally verbal children and adolescents with ASD, which are not directly attributable to autism symptom severity, intellectual disability or limitations in adaptive functioning.

## Introduction

Interest in the presence of comorbid conditions in individuals with autism spectrum disorder (ASD) has increased considerably in recent years, particularly in research aimed at characterizing the substantial phenotypic heterogeneity found in ASD and its possible underlying etiology (1–4). While many studies have documented the prevalence of various co-occurring psychiatric symptoms in children and adults with ASD, little is known about how these symptoms relate to specific phenotypic characteristics, such as expressive language ability, which varies significantly among individuals with ASD (5). When researchers have focused on particular subgroups of individuals with ASD, they were typically distinguished by the presence or absence of intellectual disability (ID), without explicit consideration of the ability to use spoken language functionally.

The majority of these studies have indicated a higher prevalence and number of different psychiatric conditions in youth with ASD compared to age-matched control groups without ASD (2, 6, 7), however the reported prevalence of these conditions has varied across studies. In a literature review of existing research on comorbid conditions in individuals with ASD, Mannion and Leader (2) attributed inconsistent findings to the use of different instruments, and differences in study participants' intellectual and communication abilities, among other factors (8–10). For instance, rates of anxiety in individuals with ASD have been estimated between 11 and 84% (11, 12), and rates of ADHD co-occurring with ASD have varied between 28 and 87% (13–15). The inconsistencies in findings across studies could be related to the diagnostic instruments commonly used to characterize psychiatric and behavior dysregulation symptoms in children and adults, which have not been specifically designed to screen for comorbid psychopathology in people with ASD [e.g., the Aberrant Behavior Checklist, ABC; (16, 17); Child Behavior Checklist, CBCL; (18); CASI-5; (19)].

Several studies have employed instruments specifically designed for use with adults with ASD [e.g., Autism Spectrum Disorders—Comorbidity for Adults, ASD-CA; (20)] or children with ASD [e.g., Baby and Infant Screen for Children with Autism Traits; BISCUIT; (21); Autism Comorbidity Interview-Present and Lifetime; ACI-PL; (22); Autism Spectrum Disorders-Comorbidity for Children, ASD-CC; (23, 24)]. Using the Autism Comorbidity Interview [ACI-PL; (22)], which was developed based on an adaptation of the Kiddie-Schedule for Affective Disorders and Schizophrenia [K-SADS; (25)], Leyfer et al. (22) found that the most prevalent psychiatric comorbidity in a sample of verbal 5-to-17-year-olds was specific phobia, present in 44% of the sample, followed by obsessive-compulsive disorder (OCD) present in 37% of the sample. In contrast, Simonoff et al. (14) reported that only 8% of their sample had a diagnosis of OCD, while the most prevalent psychiatric comorbidity was social anxiety (29%), followed by ADHD (28%) in their ASD sample between the ages of 11 and 14 years, most of whom had IQ scores over 70. The utility of these measures for assessing psychiatric comorbidities in non- or minimally verbal (MV) individuals with ASD, many of whom also have intellectual disability, is not clear, given that these studies enrolled primarily individuals with spoken language.

Thus, despite interest in the comorbid psychiatric conditions in people with ASD, there is a paucity of studies that focus on the segment of the population most severely affected, the ~30% of individuals with ASD who remain non- or minimally verbal beyond school-age (26, 27). In an effort to fill this gap, a research collaborative between several academic and medical institutions was established in 2014, with the goal of conducting common comprehensive assessments on children and adolescents with ASD admitted to inpatient psychiatry care units [the Autism Inpatient Collection; AIC; (28)]. A preliminary paper reporting on 147 participants showed that expressive language impairment (being non-, or minimally verbal) affected 52% of the sample, 43% had intellectual disability, and 27% engaged in self-injurious behavior. Elevated behavioral disturbance was common in this cohort as reflected by high average scores on the Irritability and Hyperactivity subscales of the Aberrant Behavior Checklist [ABC; (16)], which are indicative of clinically concerning externalizing symptoms (4).

A second paper based in part on the same sample (29) directly compared 165 minimally verbal individuals with ASD with a cohort of 268 verbal youth with ASD, drawn from several referral sources, mostly outpatient clinics, on the *Child and Adolescent Symptom Inventory* [CASI-4 and−5; (19, 30)] parent-report rating scales. The main findings were that, regardless of verbal ability status, inpatient participants showed greater severity and were more likely to exceed clinical cutoffs than those in outpatient settings across almost all CASI-4 and−5 psychiatric classifications. However, in comparisons between the minimally verbal and verbal groups, the verbal group had higher symptom severity, and a higher percentage of participants exceeded clinical cut-offs for depression, general anxiety disorder and oppositional defiant disorder. These results were consistent with other reports in the literature that have suggested that better functional communication was associated with higher levels of anxiety in individuals with ASD (31–34), although these other studies did not specifically enroll or investigate *minimally verbal* individuals with ASD as a group. In contrast to the profile of psychiatric comorbidities reported for verbal individuals with ASD, the MV participants assessed by Lerner et al. (29) were more likely to meet the clinical cutoff for ADHD-Hyperactivity/Impulsivity type relative to the verbal participants (50% compared to 37%), when controlling for non-verbal IQ, age, and ADOS calibrated symptom severity scores.

In sum, the majority of previous studies did not enroll non- or minimally verbal youths with ASD and only more recent studies have focused specifically on this neglected “severe end of the spectrum” (26, 28). Expanding on this research, here we examined the type, frequency, and severity of psychiatric symptoms using the CASI-5 as our primary outcome measure, and the *Emotion Dysregulation Inventory* [*EDI*; (35)], a new measure designed to assess emotion control difficulties independently of IQ and verbal ability in individuals with ASD, in a group of MV children and adolescents with ASD who have never received inpatient psychiatric care. Given that emotion dysregulation has been proposed as one potential explanatory mechanism for the high rates of psychiatric comorbidities found among individuals with ASD (36), we hypothesized that the EDI reactivity and dysphoria scales would be correlated with several of the CASI-5 symptom classifications indicative of externalizing and internalizing disorders, respectively. Because minimal capacity for spoken language is often associated with intellectual disability (ID) in ASD, we examined whether CASI-5 psychiatric comorbidities and EDI reactivity and dysphoria were associated with maladaptive behaviors in our MV-ASD sample, independently of age and ID.

The overarching aim of our study was to characterize the profile of comorbid symptoms of psychopathology and emotion dysregulation in an exclusively *outpatient* sample of children and adolescents who remained minimally verbal by school age (5 years and older). In particular, based on CASI-5 scores, we examined the severity and frequency of psychiatric symptoms in 5-to-18-year-old children and adolescents with ASD, relative to population norms. In addition, based on EDI scores, we investigated the frequency of parent-reported symptoms of emotional reactivity and dysphoria.

The specific goals of this study were:

1. To investigate whether individual differences, such as age and gender, influence the presentation of psychiatric comorbidities and emotion dysregulation among MV individuals with ASD. In particular, we examined whether children (5; 0 to 11; 11 years) differed from adolescents (12; 1 to 18; 6 years) in the type, prevalence and severity of symptoms reported by caregivers on the CASI-5 and EDI;
2. To examine the relationships between ratings of psychiatric symptoms and of emotion dysregulation and other characteristics of the sample, such as cognitive ability (IQ), adaptive functioning, maladaptive behavior, and autism symptom severity.
3. To examine whether the overall burden of psychiatric comorbidities and emotion dysregulation predict maladaptive behaviors, over and above variability in IQ and age, in MV individuals with ASD.

## Methods

### Participants

Sixty-five participants diagnosed with ASD who had limited verbal abilities (i.e., few to no words used spontaneously) were included in the study. Participants had enrolled in a larger phenotyping study of minimally verbal (MV) individuals with ASD conducted at a University-affiliated research center. They were recruited from a variety of resources in the community including schools, clinics, and social media and came from predominantly English-speaking homes, had normal or corrected-to-normal vision and hearing, and did not have significant neurological impairment. Based on a medical history survey answered by caregivers, none of the participants had ever been hospitalized in a psychiatric care unit prior to participation in our research. Informed consent was obtained from the parents, and the Boston University Institutional Review Board approved study procedures.

ASD diagnoses of the children and adolescents enrolled in the study were confirmed using the Autism Diagnostic Interview—Revised [ADI-R; (37)] conducted with primary caregivers and the ADOS. Participants aged 5 through 11 years (*n* = 33) were assessed with Module 1 of the Autism Diagnostic Observation Schedule-2 [ADOS-2; (38)]. Participants aged 12 through 18 years (*n* = 32) were assessed with Module 1 of the Adapted ADOS [A-ADOS; (39)], which uses play materials more appropriate and engaging for adolescents. The ADOS Module 1 was specifically designed to assess ASD symptomatology in children with few to no words and is therefore appropriate for defining minimally verbal ASD (40). Social-affective and restrictive and repetitive behavior symptom severity were calculated with the ADOS calibrated symptom severity scores, which are comparable across ADOS modules (41). For both the ADI-R and the ADOS assessments, higher diagnostic algorithm and calibrated symptom severity scores indicate more severe ASD symptoms. Participant characteristics are reported in Tables 1A, 1B by age group.

**Table 1A:** Participant characteristics, by age group.

	**Child**	**Adolescent**	***p^a^***
	***N*** **=** **33**	***N*** **=** **32**	
	**Mean (SD)**	**Mean (SD)**	
Age	7.59 (1.99)	14.79 (1.9)	0.001
**ADI-R SCORES**			
Social interaction	25.94 (2.72)	26.04 (3.8)	ns
Nonverbal communication	11.9 (2.22)	12.59 (1.53)	ns
Repetitive behaviors	5.35 (1.49)	5.96 (2.47)	ns
**ADOS SYMPTOM SEVERITY SCORES**			
Social affect	7.12 (1.19)	7.47 (1.74)	ns
Restricted and repetitive behaviors	8.91 (1.18)	7.94 (1.70)	0.009
Total (overall CSS)	7.70 (1.18)	7.59 (1.74)	ns
Leiter-3 nonverbal IQ	70.53 (14.69)	48.97(12.97)	0.001
**VINELAND ADAPTIVE BEHAVIOR SCALES (VABS–II)^b^**			
Communication domain	54.91 (12.26)	43.39 (9.74)	0.001
Socialization domain	55.66 (8.27)	43.85 (6.37)	0.001
Daily living skills	62.0 (10.38)	50.48 (11.02)	0.001
Adaptive behavior composite	57.13 (8.88)	44.54 (9.18)	0.001
Maladaptive behavior index^c^	19.25 (1.54)	19.14 (1.18)	ns
Internalizing behaviors^c^	19.38 (2.03)	19.64 (1.50)	ns
Externalizing behaviors^c^	17.28 (1.81)	17.79 (1.55)	ns

a Independent-samples t-test.

b Data on VABS-II was not available for 4 adolescent participants.

c *v-Scale scores*.

**Table 1B:** Demographic characteristics of the participants.

	**Child**	**Adolescent**	***P^a^***
	***N*** **=** **33**	***N*** **=** **32**	
	**Mean (SD)**	**Mean (SD)**	
Gender (Male/Female)	27/6	22/10	ns
Race/Ethnicity			ns
White	60.6%	68.8%	
Hispanic	6.0%	3.1%	
Native Hawaiian/Pacific islander	0	3.1%	
More than one race / unknown	9.1%	3.1%	
Maternal education			ns
Less than high school	0	0	
High school/GED	6.0%	6.2%	
Some college	27.3%	19.4%	
Bachelor's degree	18.2%	35.5%	
Graduate degree	45.4%	32.3%	
Other (e.g., trade vocational school)	3%	6.5%	
Household income			ns
< $50,000.00	12.12%	6.25%	
$50,000 to $100,000	12.12%	6.25%	
>$100,000.00	48.5%	53.13%	
No response	27.3%	34.37%	

a *χ^2^ test*.

### Measures and Procedure

All participants were administered a battery of cognitive diagnostic assessments and parents completed several questionnaires and interviews about their child's developmental history and current behavioral profile, either in their homes, or when their child was being tested.

### Measures of Cognitive and Adaptive Functioning

Non-verbal IQ (NVIQ) was assessed with the Leiter International Performance Scale -Third Edition [Leiter-3; (42)], a test commonly used with minimally- and low-verbal individuals with ASD (43) because it does not require verbal instructions or verbal responding. Parents completed the Vineland Adaptive Behavior Scales, Second Edition [VABS-II; (44)], a measure administered in a semi-structured interview format. In addition to assessing the level of an individual's personal and social skills required for everyday living, the VABS-II also yields a maladaptive behaviors index (including separate scores for externalizing and internalizing maladaptive behaviors), based on caregiver ratings of problematic or challenging behaviors that interfere with a person's optimal daily functioning.

### Parent-Report Measures of Comorbid Psychopathology and Emotion Dysregulation

#### Child and Adolescent Symptom Inventory (CASI-5)

We used the parent-report version of the CASI-5 to examine the frequency and severity of comorbid psychiatric symptoms in our sample. The parent version of the CASI-5 includes 173 items, which rate behaviors as occurring *never, sometimes, often and very often*. The items assess symptoms of DSM-5 psychiatric disorders, including Attention Deficit Hyperactivity Disorder (inattentive, hyperactive/impulsive, & combined types), anxiety disorders (generalized anxiety, social anxiety/'social phobia', and separation anxiety), conduct disorder and oppositional-defiant disorder, mood disorders (major depressive episode, dysthymia, and manic episode) and eating disorders (anorexia, bulimia). In addition, a limited number of symptoms characteristic of the following disorders are also included: posttraumatic stress disorder, obsessive-compulsive disorder, schizophrenia and schizoid personality, specific phobia, panic disorder, selective mutism, trichotillomania, motor tics, vocal tics, and substance use. For the majority of items, symptoms rated as occurring *often* and *very often* are considered clinically significant, and those rated as *never* or *sometimes* are not.

The CASI-5 yields several types of scores that correspond to two approaches to assessing psychiatric symptomatology. One approach is based on a dimensional scoring method that uses normative data to generate T-scores (with a mean of 50 and *SD* = 10) for each symptom classification based on the participant's gender and age. For the purposes of this study, we considered severity T-scores > 65 (i.e., > 1.5 SD above the mean and at the 93rd percentile or higher relative to the norm sample) as clinically and functionally significant. The second approach to scoring is categorical and involves determining whether an individual meets criteria for a particular DSM-5 screening diagnosis based on the number of clinically concerning symptoms shown (e.g., items rated *often and very often*); this number—labeled S*ymptom Count Score*—is compared to a *Symptom Count Cutoff* **/**criterion score for each disorder classification. It should be noted that symptom count cutoff scores do not indicate a psychiatric diagnosis, and their relevance is restricted to screening purposes for clinically concerning symptoms related to a specific disorder. According to recent reviews [e.g., (45)], the CASI-5 subscale scores generally show a high degree of correspondence with psychiatric diagnoses (predictive validity) and correlate well with other commonly used dimensional scales (concurrent validity), demonstrating satisfactory psychometric properties for a diversity of youth, including those with ASD (30).

#### Emotion Dysregulation Inventory (EDI)

Emotion dysregulation was assessed with the 66-item version of the EDI, a caregiver-report questionnaire designed to capture emotional distress and a wide range of problems with emotion regulation in youth with ASD ages 6 years and above. The items describe observable indicators of poor emotion regulation, which are rated by caregivers on a 0 to 4 scale from “*not at all—never happens”* to “*very severe—almost always happening and causes a serious problem.”* The EDI is comprised of two scales: a *Reactivity scale*, which captures intense, rapidly escalating, sustained, and poorly regulated negative emotional reactions, and a *Dysphoria scale*, characterized by minimal positive affect and motivation, and the presence of nervousness and sadness. The EDI reactivity and dysphoria scales yield raw scores that were converted into T-scores based on tables provided to us by the instrument's author. It should be noted that the calibration sample for this instrument consisted of a large combined sample of 1,751 community and psychiatric inpatients with ASD. Therefore, the results we present are relative to the autism norms and scoring that were validated for this population (36).

### Statistical Analysis

Descriptive statistics were calculated using means and standard deviations for continuous variables and frequencies and proportions for categorical variables. First, children and adolescent groups were compared on demographic variables, including scores on standardized tests of cognitive and adaptive functioning and autism symptom severity, using independent sample *t*-tests or χ^2^ tests, as appropriate. Variables on which significant group differences were found (e.g., NVIQ) were controlled for (entered as covariates) in analyses of the CASI-5 and EDI factors variables, conducted to compare psychiatric comorbidities and emotion dysregulation ratings across the age groups.

We conducted several types of analyses of the CASI-5 parent ratings to characterize the profile of psychiatric comorbidities in our sample. First, we examined the prevalence of different types of comorbidities based on the frequencies of scores exceeding the clinical cut-off for each CASI-5 classification (categorical scoring approach). Then we explored the distribution of clinically significant CASI-5 symptom severity scores (i.e., T-score > 65) in our sample relative to the distribution expected in the general population, using χ^2^ to test for significant differences. We further examined age and gender group differences in the severity of comorbid symptoms (dimensional scoring) using a multivariate analysis of variance approach with the CASI-5 T-scores as the dependent variables, while co-varying NVIQ. More specifically, the continuous CASI-5 *symptom severity* variables (T-scores) for the psychiatric classifications relevant for both age groups (nine classifications or subscales) were entered into a multivariate analysis of covariance (MANCOVA) to determine if symptom severity-scores across classifications differed as a function of age-group and gender, after controlling for NVIQ. To further examine group differences, the multivariate test was followed by univariate ANOVAs and *post-hoc* tests, as needed, using Bonferroni corrected significance levels. A similar MANCOVA was conducted with the two EDI factors as dependent variables, age-group and gender as between-subjects factors and NVIQ as a covariate, to examine possible group differences in aspects of emotion dysregulation, after controlling for cognitive ability. Relationships between psychiatric comorbidities, emotion dysregulation factors and level of cognitive functioning (NVIQ), were examined using Pearson correlations. We further investigated relationships between selected psychiatric comorbidities, emotion dysregulation factors, autism symptom severity and ratings of maladaptive behavior on the VABS-II while controlling for NVIQ, using partial correlations and the Holm-Bonferroni method to correct for multiple testing. Finally, to investigate if psychiatric comorbidities or emotion dysregulation factors contributed significantly to ratings of maladaptive behavior on the VABS-II, a stepwise multiple regression analysis was conducted, entering age and NVIQ on the first step, followed by the number of clinically significant comorbidities endorsed by parents on the CASI-5 and the two EDI emotion dysregulation factors as an independent variables, with VABS-II Maladaptive behavior index scores as the dependent variable.

## Results

Tables 1A,B present demographic characteristics of the participants, by age group. No differences were found between males and females on any characteristic listed. The child and adolescent groups did not differ in gender, race/ethnicity, parent education, and household income distributions (based on χ^2^ tests, all *p* > 0.25), or on ADI-R or ADOS overall calibrated symptom severity scores. Independent-samples *t*-tests showed that younger participants obtained, on average, higher standard scores on several measures of cognitive and adaptive functioning than the adolescent group (see Table 1A). Non-verbal IQ and VABS-II Adaptive Behavior Composite scores were highly correlated (*r* = 0.782, *p* < 0.0001), even when adjusting for age differences (*r*_*p*_ = 0.649, *p* < 0.0001). Therefore, all analyses of group differences on the variables of interest from the CASI-5 and the EDI survey were conducted co-varying NVIQ.

### Sample Characterization With the CASI-5

**Figure 1:**
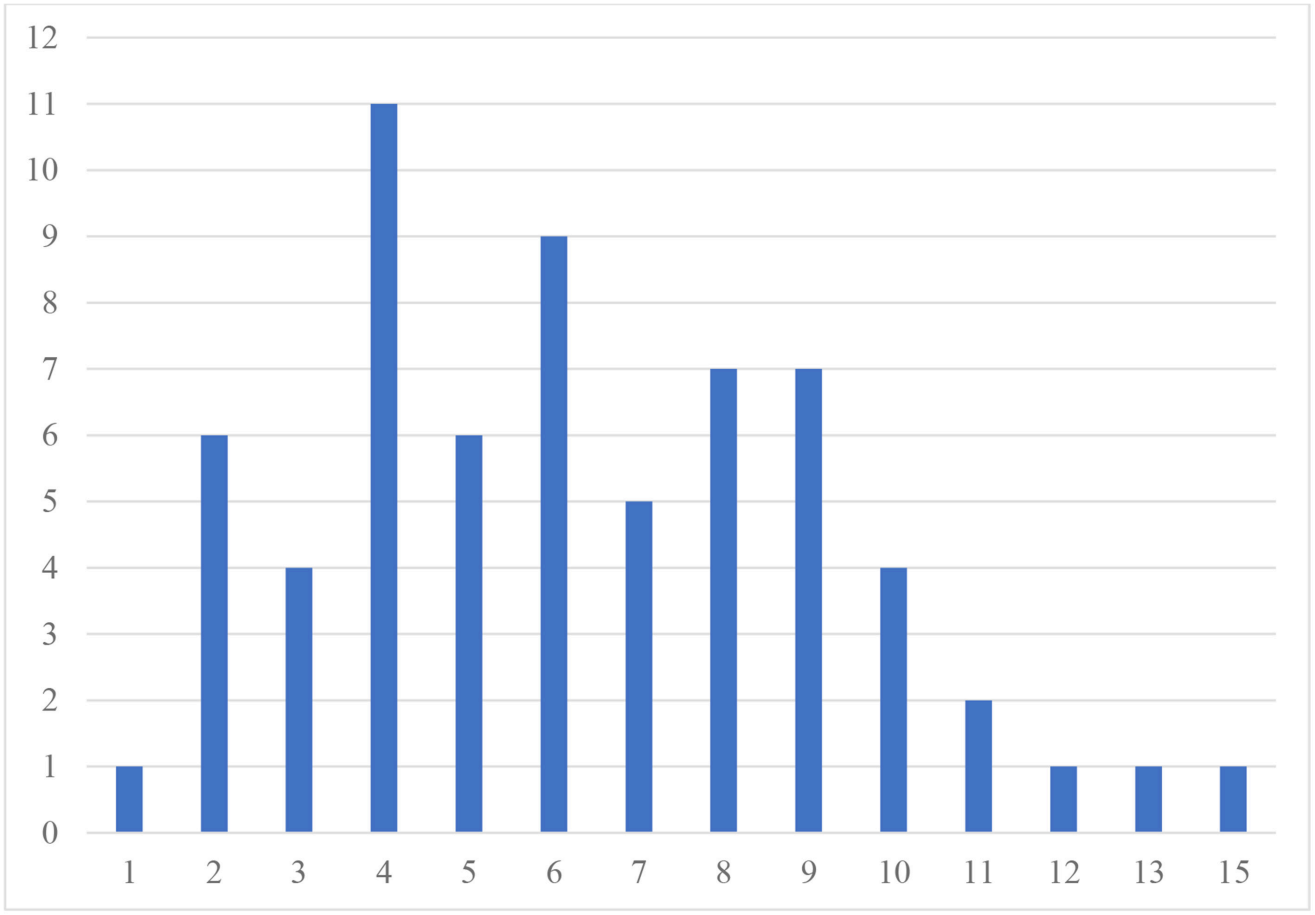
Frequency of participants by number of CASI−5 symptom classifications with clinically significant ratings. Number of psychiatric comorbidities exceeding clinical cutoff scores.

Table 2 presents the prevalence of different psychiatric comorbidities in our sample for children and adolescents, based on the CASI-5 categorical scoring, which takes into account whether an individual meets criteria for a particular DSM-5 diagnosis based on the number of clinically concerning symptoms shown (i.e., exceeds a *Symptom Count Cutoff* criterion for a particular CASI-5 classification). All participants met cutoff criteria for at least one CASI-5 classification, and the number of categorical classifications parents endorsed ranged from 1 to 15, with a mode and a median of 6 classifications. Figure 1 shows the distribution of participants by the number of symptom classifications on which they met clinical cutoff criteria on the CASI-5.

**Table 2:** Prevalence of participants meeting clinical cut-off scores on CASI-5 symptom classifications.

**N**	**Children**	**Adolescents**	**Entire sample**
	**33**	**32**	**65**
	**% of sample**	**% of sample**	**% of sample**
**EXTERNALIZING DISORDERS**			
ADHD			
Inattentive type	48.5	31.25	40
Hyperactive/impulsive type	21.21	25	23.1
Combined type	18.18	18.75	18.5
Oppositional defiant disorder	3.03	3.13	3.1
Conduct disorder	3.03	6.25	4.6
**INTERNALIZING DISORDERS**			
Generalized anxiety disorder	3.03	3.13	3.1
Major depressive disorder	0	3.13	1.5
Dysthymic disorder	3.03	3.13	3.1
Social phobia/social anxiety	12.12	6.2	9.2
Separation anxiety disorder	3.03	3.03	3.1
**OTHER DISORDERS**			
Specific phobia	45.5	40.6	43.1
Panic disorder	0	0	0
Obsessions	6.06	3.13	4.6
Compulsions	36.4	31.25	33.8
Posttraumatic stress	15.2	12.5	13.8
Motor tics	42.4	50	46.2
Vocal tics	54.6	78.13	66.2
Somatic symptoms	3.03	0	1.5
Enuresis	60.6	6.06	52.3
Anorexia nervosa	3.03	3.13	3.1
Bulimia nervosa	6.06	25	15.4
Schizoid personality disorder	12.1	28.1	20
Schizophrenia	0	0	0
Bipolar disorder/Manic episode	3.03	3.13	3.1

The most frequent symptom classification endorsed by parents in our sample was *vocal tics*, with 66.2% of the sample meeting clinical cutoff. Forty-six percent of the sample met the clinical cutoff for *motor tics*, followed by *specific phobia*, reported to be present in 43% of the sample. A large proportion of the sample met the clinical cutoff for ADHD Inattentive type (40%), whereas 23% met clinical cutoff for the ADHD Hyperactive-impulsive type, followed by ADHD Combined type (18.5%). Compulsions were reported in about a third of the sample. However, except for vocal tics, no single psychiatric symptom was found significantly elevated in more than 50% of the sample, reflecting the wide variety and complex combinations of clinically significant psychiatric symptoms among our MV participants. The one exception was enuresis, which was reported for 60.6% of children but only for 6% of adolescents, suggesting that this comorbid condition is likely to resolve over time.

We also characterized the profile of psychiatric comorbidities in this population based on analyses of symptom severity, afforded by the dimensional scoring approach for the CASI-5 classifications. Symptom severity scores on the CASI-5 indicate whether an individual shows symptom levels of clinical concern, even if not having the number of symptoms required to screen positive for a DSM-5 psychiatric diagnosis. Therefore, we examined the distribution of *T*-scores in our sample, taking as a threshold for clinical significance *T*-scores higher than 1.5 standard deviation from the mean (i.e., *T*-scores of 65 and above). Table 3 shows the percentage of participants who obtained *T*-scores above and below 65. When comparing this distribution of scores to the normal distribution, we found that for all symptom categories, except oppositional defiant disorder and conduct disorder, more participants showed clinically concerning severity scores than expected based on general population norms.

**Table 3:** Proportion of participants (%) by Distribution of clinically significant T-scores on the CASI-5.

**T-score**	** < 65**	**>65**	**χ^2^**	***p***
Normal distribution %	93%	7%		
**EXTERNALIZING DISORDERS**				
ADHD				
Inattentive type	55.4	44.6	141.3,	0.0001
Hyperactive/impulsive type	52.3	47.7	165.3,	0.0001
Combined type	44.6	55.4	233.7,	0.0001
Oppositional defiant disorder	96.9	3.1	1.54,	ns
Conduct disorder	96.9	3.1	1.54,	ns
**INTERNALIZING DISORDERS**				
Generalized anxiety disorder	72.3	27.7	42.75,	0.0001
Depressive disorders				
Major depressive disorder	80	20	16.87,	0.0001
Dysthymic disorder	83.1	16.9	9.83,	0.002
Social phobia^a^	72.1	27.9	28.9,	0.0001
Separation anxiety disorder	84.6	15.4	7.02,	0.008
**OTHER DISORDERS**^b^				
Schizophrenia	58.6	41.4	52.6,	0.0001
Schizoid personality disorder	38.7	61.3	140.4,	0.0001
Bipolar disorder/Manic episode	81.3	18.8	6.79,	0.009

a Classification T-score applies only to children (5–12 years).

b *Classification T-scores apply only to adolescents (12–18 years)*.

Symptom severity ratings of psychiatric comorbidities (CASI-5 T-scores) for the 9 symptom classifications common to children and adolescents (see Table 4) were entered into a MANCOVA with age-group and gender as between-subjects factors and NVIQ as covariate. This analysis yielded a significant multivariate effect of age group, *F*_(9, 51)_ = 3.291, *p* = 0.003, Wilks' Λ = 0.633, partial η^2^ = 0.367, with no other significant effects or interactions. Follow-up univariate ANOVAs indicated that children and adolescents differed significantly in the severity of their symptoms for ADHD Inattentive type, *F*_(1, 59)_ = 7.10, *p* = 0.01, partial η^2^ = 0.11, with the children showing more impairment than the adolescents (mean = 67.9, *SD* = 14.9 vs. mean = 63.7, *SD* = 12.3, respectively) and on Major depressive episode, *F*_(1, 59)_ = 6.32, *p* = 0.015, partial η^2^ = 0.10, with the adolescents (mean = 61.97, *SD* = 13.6) showing more impairment than the children (mean = 52.06, *SD* = 9.9) in this category of psychiatric comorbidity.

**Table 4:** Mean (and Standard Deviations) of CASI-5 severity scores (T-scores) for symptom classifications.

	**Child**	**Adolescent**	p^*^
	**Mean (SD)**	**Mean (SD)**	
**DISRUPTIVE BEHAVIOR DISORDERS**			
ADHD			
Inattentive type	67.85 (14.86)	63.75 (12.31)	0.01
Hyperactive/impulsive type	62.12 (10.72)	69.19 (11.17)	ns
Combined type	67.0 (13.58)	67.8 (12.82)	ns
Oppositional defiant disorder	45.24 (5.96)	47.09 (11.21)	ns
Conduct disorder	46.82 (2.8)	48.84 (7.38)	ns
**ANXIETY DISORDERS**			
Generalized anxiety disorder	57.24 (9.06)	58.13 (10.9)	ns
Separation anxiety disorder	50.18 (8.69)	57.59 (16.67)	ns
**MOOD DISORDERS**			
Major depressive episode	52.06 (9.89)	61.97 (13.63)	0.015
Dysthymic disorder	53.23 (10.05)	61.47 (15.67)	ns

* *Pairwise comparisons among estimated marginal means, controlling for NVIQ*.

### Sample Characterization on the EDI

Table 5 presents the T-scores for the children and adolescents in our sample on the EDI reactivity and dysphoria scales. A MANCOVA with EDI reactivity and dysphoria T-scores as dependent variables, age-group and gender as between-subjects factors, and NVIQ as a covariate did not yield any main effects or interactions indicating that, in our sample, severity of emotion dysregulation was largely independent of age-group, [*F*_2, 49_ = 1.54, *p* = 0.224, Wilks' Λ = 0.941, partial η^2^ = 0.06], gender [*F*_2, 49_ = 0.953, *p* = 0.392, Wilks' Λ = 0.963, partial η^2^ = 0.037], or NVIQ [*F*_2, 49_ = 0.761, *p* = 0.473, Wilks' Λ = 0.796, partial η^2^ = 0.03].

**Table 5:** Means (and Standard Deviations) of T-scores on EDI factors by age-group.

	**Child**	**Adolescent**	p^*^
	**Mean (SD)**	**Mean (SD)**	
**EMOTION DYSREGULATION (EDI)**			
Reactivity^a^	41.91 (4.94)	43.65 (6.51)	ns
Dysphoria^a^	42.13 (4.96)	43.93 (6.30)	ns

a T-scores.

* *Pairwise comparisons between estimated marginal means, controlling for NVIQ*.

Overall, relative to the normative autism calibration sample, the participants in this study did not show significantly elevated symptoms of reactivity or dysphoria on the EDI, according to parent report for current behaviors. The range of T-scores was 30 to 60 for reactivity and 36 to 58 for dysphoria in our sample, suggesting that few caregivers rated behaviors indicative of emotion dysregulation as *very severe problems*, although across participants the full scale (0 to 4) was used for most items. Some items included in the dysphoria scale, however, never received a rating of *severe* or *very severe* (e.g., “seems sad or unhappy,” “appears uneasy through the day”).

### Relationships Among Parent-Report Measures of Psychiatric Symptoms, Emotion Dysregulation, IQ, Adaptive Functioning, Maladaptive Behaviors, and Autism Severity

First, we examined correlations between cognitive functioning (NVIQ) and severity scores for the CASI-5 classifications and the EDI factors. NVIQ was significantly negatively correlated with CASI-5 T- scores only for symptoms of ADHD (for ADHD Hyperactivity-impulsive type, *r*_(64)_ = −0.381, *p* = 0.002, and *r*_(64)_ = −0.274, *p* = 0.024 for ADHD Combined type). No other psychiatric comorbidities rated on the CASI-5 correlated significantly with NVIQ. On the EDI, neither the reactivity nor the dysphoria factors were correlated with NVIQ. However, because the two age groups differed significantly in NVIQ scores, we conducted all other correlational analyses controlling for NVIQ. Adaptive functioning scores (VABS-II Adaptive Behavior Composite scores) were not correlated with any CASI-5 severity T-scores or with the EDI factors in our sample.

Next, we examined autism symptom severity scores as related to measures of psychiatric comorbidities and emotion dysregulation severity, controlling for NVIQ. We found no significant correlations between T- scores on the CASI-5 or the EDI factors and ADOS calibrated severity scores. To investigate whether psychiatric comorbidities and emotion dysregulation contributed to behavioral dysregulation as assessed by ratings of maladaptive behaviors (i.e., internalizing and externalizing behaviors) on the VABS-II, we examined correlations between CASI-5 and EDI factors T-scores, and VABS-II indices of internalizing and of externalizing behaviors, controlling for NVIQ. We selected from the CASI-5 the symptom classifications for which T-scores are provided for both children and adolescents, and for which our sample showed elevated severity (T scores > 65, cf. Table 3). Table 6 presents partial correlations among our primary measures from the CASI-5 and the EDI, and indices of maladaptive behavior from the VABS-II, controlling for NVIQ. As expected based on the theoretical model of emotion dysregulation underlying the EDI, the reactivity factor index on the EDI was significantly correlated with ratings of externalizing behaviors on the VABS-II after controlling for cognitive ability, *r*_*p*__(53)_ = 0.383, *p* = 0.006. The dysphoria factor, however, was not significantly correlated with internalizing behaviors scores on the VABS-II. While EDI reactivity and dysphoria T-scores were both correlated with the number of clinically significant CASI-5 symptom classifications (a measure of “psychiatric burden”), only the EDI dysphoria factor was significantly correlated with the generalized anxiety CASI-5 classification, *r*_*p*_(54) = 0.398, *p* = 0.002, after adjusting for multiple testing (Table 6).

**Table 6:** Partial correlations between severity scores for selected psychiatric symptom classifications (CASI-5), emotion dysregulation (EDI) factors, and maladaptive behavior (VABS-II), controlling for cognitive ability (NVIQ).

	**VABS-II internalizing behavior**	**VABS-II externalizing behavior**	**EDI reactivity**	**EDI dysphoria**	**Number of comorbid symptoms**
ADHD inattentive type	0.202	0.269	0.060	0.267	0.440^*^
ADHD hyperactive /impulsive type	0.113	0.474^*^	0.214	0.181	0.425^*^
ADHD combined type	0.186	0.385^*^	0.129	0.274	0.472^*^
Generalized anxiety disorder	0.284	0.342	0.298	0.398^*^	0.577^**^
Major depressive disorder	−0.228	−0.056	0.209	0.302	0.342^*^
Number of comorbid symptoms	0.245	0.244	0.407^*^	441^*^	
EDI Dysphoria	0.100	0.076	0.554^**^		
EDI Reactivity	0.079	0.383^*^			

Significance level after Holm-Bonferroni correction:

* p < 0.001;

** *p < 0.0001*.

Finally, we were interested in exploring to what extent the burden of psychiatric comorbidities and emotion dysregulation predicted caregivers' ratings of maladaptive behaviors in their children, as reported on the VABS-II maladaptive behavior index. To this end, we conducted a stepwise multiple regression analysis entering chronological age and NVIQ on the first step, followed by entering the two EDI factors (dysphoria and reactivity) and the total number of clinically significant symptom classifications from CASI-5 on the second step. Because neither the ASD symptom severity calibrated score, nor the overall adaptive functioning measure (VABS-II Adaptive Behavior Composite) were correlated with any of the psychiatric comorbidity or emotion dysregulation T- scores in our sample, we did not enter these variables in the analysis. Results revealed that, when entered in stepwise fashion, only the number of comorbid symptom classifications on the CASI-5 was retained as predictor, explaining 9.5% of variance in the VABS-II maladaptive index scores [adjusted *R*^2^ = 0.095; *F*_1, 50_ = 5.22. *p* = *0*.027], whereas the EDI factors did not make a significant contribution to the model (EDI reactivity β = 0.125, t = 0.829, ns, and EDI dysphoria β = −0.092, t = −0.610, ns).

## Discussion

In this first study of a relatively large and never hospitalized outpatient sample of minimally verbal children and adolescents with ASD, our main goal was to investigate co-occurring psychiatric symptoms using several different measures to provide a comprehensive phenotypic characterization of MV individuals as related to psychiatric and emotion dysregulation symptomatology. We found high rates of psychiatric symptomatology on the CASI-5, but relatively low rates of emotional dysregulation, especially dysphoria on the EDI. We also found that the number of different psychiatric symptom classifications endorsed on the CASI-5 was a key predictor of maladaptive behavior. The overall picture to emerge from this study is that minimally verbal children and adolescents present with extremely heterogeneous profiles of co-morbid psychopathology that are not easily predicted by autism symptom severity, intellectual disability, or limitations in communication.

Our main analyses focused on the CASI-5, a well-validated parent-report psychiatric screening measure. Perhaps the most striking finding was that virtually every participant met cut-off criteria for at least one co-morbid condition, and the average number of different co-morbidities across the sample was six. The most common conditions included tics and phobias and among psychiatric categories, ADHD was the most common. In contrast, we found low rates of oppositional defiant disorder and conduct disorder, perhaps reflecting the limited opportunities for exhibiting signs of these disorders in the population we studied, or caregivers' construal of their children's behavior. In general, our findings are consistent with other reports in the literature. In particular, the pattern of psychopathology we found matches what Lerner and his colleagues reported (29) in their sample of minimally verbal youth with ASD, although the rates that we found were lower. This is not surprising since our sample had never been hospitalized whereas Lerner et al.'s minimally verbal participants were drawn largely from a current inpatient sample. Finally, we note that on the CASI-5, as well as our other measures of co-morbid psychopathology, we found very few differences between children and adolescents. Rates of different disorders as well as the number of different conditions were similar across the sample. We also did not find differences between males and females, although the small number of females who provided data in this study precludes drawing valid conclusions about gender-related similarities or differences in the profile of psychiatric comorbidities of MV individuals with ASD.

Our second key measure for assessing psychopathology in this study was the newly developed instrument, the EDI. This measure, which taps two different emotion dysregulation factors, reactivity and dysphoria, was specifically designed for and normed on an ASD sample (35, 36). In contrast to the significant psychopathology reported on the CASI-5, we found relatively low rates of clinically significant dysregulation compared to the instrument norms, with rates for dysphoria especially low. One reason for our lower rates might again be related to the difference in our sample since Mazefsky and colleagues normed the EDI on a large sample of both inpatient and outpatient children and adolescents with ASD and included both verbal and minimally verbal individuals. Since rates of certain co-morbid psychopathological conditions, particularly anxiety, depression and ODD are significantly more prevalent among more verbal individuals, and the EDI is correlated with CASI-5 psychopathology, it is likely that our EDI findings reflect the lower end of the ASD distribution of scores on this instrument. A second explanation for the lower EDI scores in our sample may be that parents either have trouble discerning the internal emotional states of their minimally verbal children, or that they interpret their child's behavior and affect more in terms of their primary diagnosis of ASD coupled with their severely limited communicative abilities. Thus, if a child cannot say, for example, they are unhappy or do not want to go to school, parents fail to interpret behaviors that are consistent with these emotional states in this way and therefore do not endorse those items on the EDI.

We investigated the relationship between comorbid psychopathology and other behavioral characteristics, including non-verbal IQ, adaptive functioning, and autism symptom severity. Among minimally verbal children and adolescents, autism severity scores were not related to any of our measures of psychopathology and even IQ and adaptive behavior scores were either not related or only to a modest degree (with *r*-values all below 0.4). The lack of correlations between CASI-5 scores and a general measure of adaptive functioning (VABS-II composite scores) may seem surprising, in light of findings from other research that documented associations between co-occurring psychopathology and adaptive behavior in individuals with ASD (32, 46, 47). However, these findings were based primarily on samples of individuals with ASD without intellectual disabilities (46, 48–51) or on mixed samples including both individuals with IQ in the average range and those with ID, but whether the mixed samples included MV-ASD participants remained unspecified [see (52) for a review]. In the few studies that focused on individuals with ASD and ID (53, 54) researchers examined primarily associations between psychiatric comorbidities and “problem behaviors” that directly impact adaptive functioning. In our MV-ASD sample we also found expected associations between psychiatric burden tapped by CASI-5 scores, the Dysregulation factor of the EDI and maladaptive behaviors on the VABS-II.

Consistent with the underlying model for the EDI, we found correlations between the CASI-5 and the EDI. Both EDI factors correlated significantly with most of the CASI-5 symptom classifications severity scores and with the overall number of different CASI-5 symptom classifications endorsed by each respondent. Nevertheless, in our regression analysis exploring which variables were the strongest concurrent predictors of overall maladaptive behavior, only the number of different CASI-5 symptom classifications accounted for 9.5% of the variance; Although this is not a large portion of the variance, it was significant in the context of model tested, whereas no other predictor variables, including any of the EDI factors, was significant. In some respect then, the number of different symptom classifications may function as a cumulative risk index for maladaptive behavior. Future research should explore further whether models of this cumulative risk of co-morbid psychopathology for minimally verbal individuals with ASD would be more sensitive if certain comorbid conditions were weighted higher than others were, but this would require a significantly larger sample of individuals than we had available. It would also be important to begin exploring whether there are other factors that may protect some children and adolescents from higher levels of maladaptive behavior, despite carrying a significant burden of co-morbid psychopathology.

There are some notable limitations to this study. Most importantly, we relied exclusively on parent report questionnaires, which are generally less accurate than either in-depth caregiver interviews or direct observation and evaluation of psychiatric conditions. Since minimally verbal individuals are, by definition, not able to report on their own feelings and behavior, even a direct evaluation would depend largely on parent report coupled with observations in a clinical setting. Still, our findings may have been enhanced had they been coupled with such observations carried out by an expert clinician. We focused here specifically on an outpatient group of minimally verbal children and adolescents, thus complementing the work reported by Lerner and his colleagues (26; see also 33). Because of the nature of the larger lab-based research study in which the participants were enrolled, we excluded those with the most severe behavior problems including aggression, self-injury or non-compliance, and therefore our findings must be viewed in the context of whom our participants represent. Nevertheless, this study is an important step forward in work that characterizes the minimally verbal end of the autism spectrum who have so often been excluded from earlier studies (cf. 24).

In sum, our study highlights the wide-ranging profiles of comorbid psychopathology and degree of maladaptive behavior among minimally verbal children and adolescents with ASD. The findings do not suggest that the presence of these comorbidities represent a subtype within the ASD population; on the contrary, comorbid psychopathology is the norm rather than the exception. This suggests that beginning at an early age every minimally verbal person with ASD should have ongoing clinical diagnostic and treatment services that focus on these comorbid conditions, which may well change over time within each child. It is likely that the burden of care for minimally verbal people is related as much to their comorbid conditions, including both the absence of functional language and psychopathology. As we work toward including this end of the spectrum into our fuller understanding of ASD, we need to embrace the highly complex and unique behavioral profiles of each individual.

## Author Contributions

HT-F, DPS, and RJ conceived of the study and wrote the manuscript. DPS, BE, and SM collected, coded and analyzed the data. RJ contributed to data analysis. BE and SM reviewed and approved the manuscript.

### Conflict of Interest Statement

The authors declare that the research was conducted in the absence of any commercial or financial relationships that could be construed as a potential conflict of interest.
